# Advances in 3D bioprinting for medical application: opportunities and challenges

**DOI:** 10.1186/s12938-025-01498-y

**Published:** 2025-12-16

**Authors:** Mahdi Kazemi, Sepideh Maralbashi

**Affiliations:** 1 Department of Mechanical Engineering, Isfahan University of Technology, P.O. Box: 8415683111, Isfahan, Iran; 2 Department of Immunology, Faculty of Medicine, Tabriz University of Medical Science, Tabriz, Iran

**Keywords:** Tissue engineering, 3D bioprinting, Regenerative medicine, Bioinks, Clinical translation, Vascularization, Bioprinted organs

## Abstract

Advances in 3D bioprinting technology are increasingly shaping medical applications, offering practical opportunities in tissue engineering, regenerative medicine, and personalized healthcare. By enabling the precise deposition of cells and biomaterials, 3D bioprinting allows the fabrication of functional, tissue-like constructs that reproduce key aspects of native human organs. Concrete progress has been demonstrated in applications, such as cartilage repair, skin grafts, and liver tissue models, which illustrate the translational potential of this technology. In addition, 3D bioprinted constructs are being explored for organ transplantation, drug testing, and disease modeling, where they can provide more physiologically relevant data than traditional models. Despite these advances, major challenges remain, including vascularization, mechanical stability, and ensuring long-term tissue functionality. The development of robust bioinks, regulatory acceptance, and the high cost of bioprinting platforms also represent significant barriers to widespread clinical adoption. This article reviews both the opportunities and challenges of 3D bioprinting in medicine, highlighting recent technological progress, ongoing preclinical research, and potential strategies for overcoming current limitations to accelerate clinical translation. Ultimately, 3D bioprinting is moving from proof-of-concept studies toward early clinical applications, underscoring its potential to become a transformative tool in regenerative medicine.

## Introduction

Three-dimensional (3D) bioprinting has emerged as a transformative technology in tissue engineering, reshaping how tissues and organs are fabricated. Using additive manufacturing (AM) principles, the process combines living cells, biomaterials, and bioactive molecules into functional architectures designed to mimic native tissue [1, 2]. Recent advances in 3D bioprinting have enabled the creation of patient-specific constructs for regenerative medicine and pharmaceutical testing, addressing the urgent global shortage of transplantable organs [3].

3D bioprinting employs computer-aided design (CAD) to precisely deposit bioinks—mixtures of cells, hydrogels, and growth factors—allowing fabrication of complex biological structures with high spatial control [4–6]. These constructs can potentially reduce immune rejection and improve integration with host tissue [7, 8]. As a multidisciplinary field spanning biology, materials science, and informatics, bioprinting integrates techniques, such as decellularization–recellularization and vascularized tissue fabrication [9–11].

Recent studies highlight the rapid evolution of bioink materials, printing strategies, and hybrid fabrication systems [12–16]. Despite remarkable progress, challenges remain in achieving full vascularization, functional maturation, and large-scale clinical translation. This review summarizes recent advances in 3D bioprinting for medical applications, emphasizing opportunities, technical challenges, and future directions toward clinically viable bioprinted tissues and organs. Several recent reviews have highlighted the rapid progress of 3D bioprinting in regenerative medicine and organ mode. However, a comprehensive synthesis of emerging technologies, clinical translation challenges, and future opportunities is still required, which forms the focus of this review.

## Techniques of 3D bioprinting in medicine

3D Bioprinting employs various techniques, each with unique capabilities. Extrusion 3D bioprinting technique remains a staple for large-scale tissue fabrication, while laser-assisted 3D bioprinting techniques offer the precision needed for microvascular networks. Emerging processes like projection-based 3D bioprinting achieve high-resolution models, bridging the gap between research and clinical applications. Together, these various techniques drive progress in creating functional and transplantable tissues [17]. The types of 3D printing methods used in this field are as follows:

### Extrusion 3D bioprinting

**Fig. 1:**
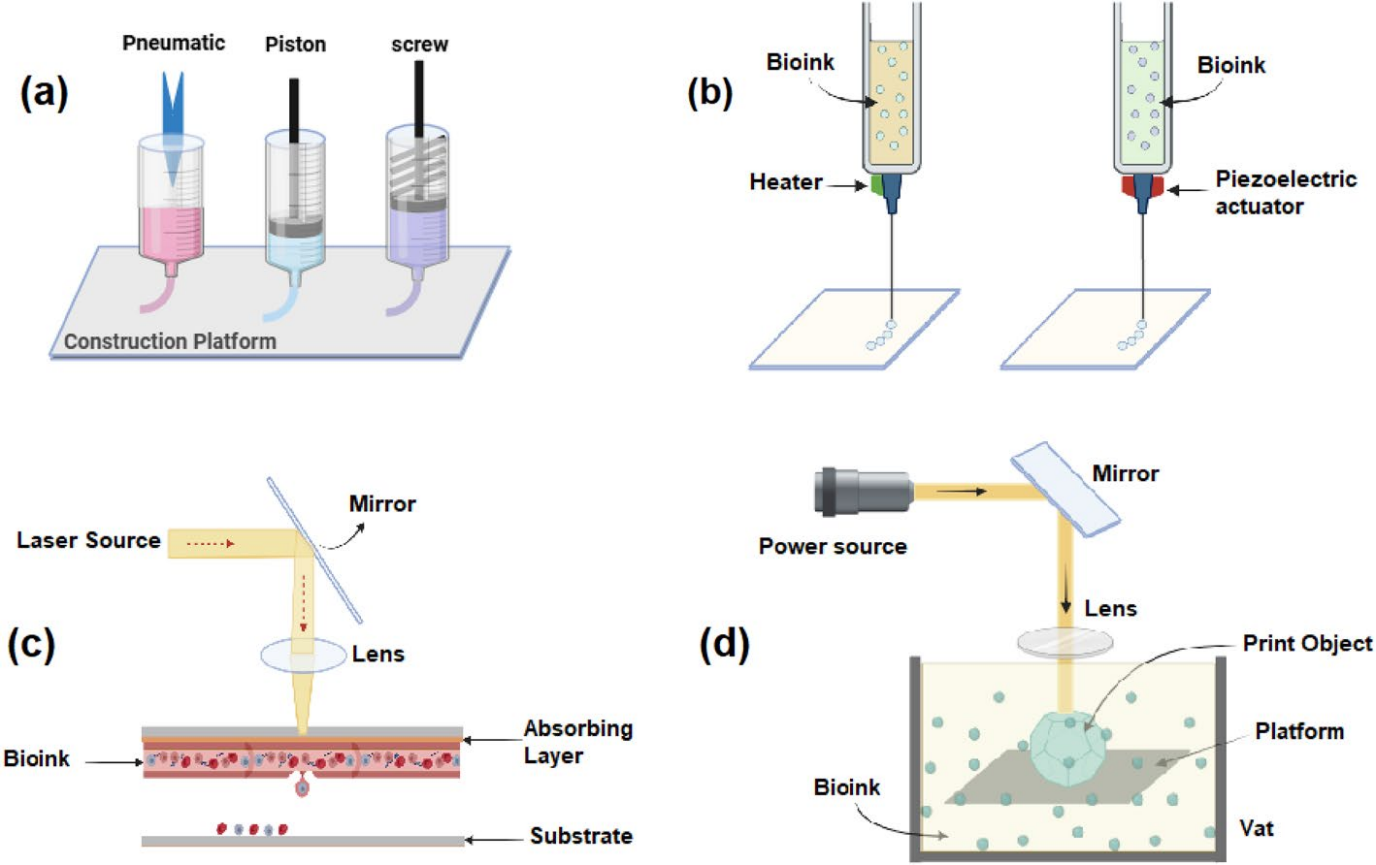
3D bioprinting techniques in medicine. **a** Extrusion 3D bioprinting. **b** Inkjet 3D bioprinting. **c** Laser-assisted 3D bioprinting. **d** stereolithography 3D bioprinting

This technique is one of the most widely used 3D bioprinting methods. It works by extruding bioinks (mixture of biomaterials and living cells) through a nozzle, thereby constructing tissues layer by layer. The resulting structures, as shown in Fig. 1a, demonstrate the precise spatial arrangement achievable with this method, highlighting its importance for tissue engineering applications. This method is frequently used for creating large-scale tissue constructs, such as bone or cartilage [18]. It is particularly useful for vascularization, since it allows the deposition of multiple cell types, including endothelial cells for vascular networks [19]. The resolution of extrusion printing is generally lower compared to other techniques, and the shear forces generated during extrusion can damage sensitive cells. Malekpour et al. [20] demonstrated that critical parameters, including needle type and size, bioink concentration, and dispensing pressure, significantly influence cell viability by contributing to mechanical stress and potential cell damage. While extrusion is versatile in bioink selection, inkjet 3D bioprinting offers greater resolution but is limited to low-viscosity inks.

### Inkjet 3D bioprinting

Inkjet 3D bioprinting, depicted in Fig. 1b, ejects tiny bioink droplets, allowing precise placement of cells for building complex tissue patterns. This technique is highly precise and efficient. Inkjet 3D bioprinting is effective for creating detailed patterns of vascular tissues, such as capillaries and arteries, by printing endothelial cells and growth factors in a controlled manner. It is limited to low-viscosity bioinks and often struggles with printing bioinks with high viscosity or larger tissues [21]. Kumar et al. [22] highlighted that inkjet printing, owing to its high resolution and precision, is a highly suitable technique for bottom-up cell deposition, enabling the fabrication of intricate biological constructs, including both 2D and 3D structures when combined with appropriate bioinks. Unlike inkjet, which relies on droplet deposition, laser-assisted 3D bioprinting achieves even higher precision but involves higher costs and complex setup.

### Laser-assisted 3D bioprinting (LAB)

Laser-assisted 3D bioprinting (Fig. 1c) uses a focused laser to deposit bioink onto a substrate with high precision, enabling accurate cell placement. It can print extremely fine details and precise structures. This technology is particularly effective for printing microvascular networks and fine cellular patterns required for vascularization in tissue engineering. It is also used for high-resolution vascular models [23]. Recent advancements combine LAB with bioactive molecules, enabling the creation of neural tissues that replicate the architecture of human brain. This precision is crucial for complex organ systems, such as the heart and brain. It requires specific bioinks and high precision, which leads to higher costs [24]. Hall et al. [23] developed a laser-assisted 3D bioprinting approach utilizing multicellular spheroids as building blocks, demonstrating that human perivascular-derived cell spheroids with diameters ranging from 100 to 300 µm can be successfully 3D bioprinted without compromising cell viability or the formation of cartilaginous extracellular matrix over 14 day post-3D bioprinting. Although laser-assisted bioprinting achieves very high precision and cell viability, the complexity and cost of this method limit its widespread use; consequently, stereolithography has emerged as another light-based approach that provides high resolution with comparatively simpler operational requirements.

### Stereolithography (SLA)

Stereolithography (Fig. 1d) uses UV light to cure photosensitive bioinks layer by layer, forming the desired 3D structures. This method is known for its high resolution and ability to print intricate models [25]. SLA is particularly useful for creating small-scale vascular networks and neural tissue with high precision. It is suitable for vascular scaffolding and tissues requiring fine and detailed vascular networks. This technique requires UV-sensitive bioinks, which may not be ideal for certain cell types, and there are challenges with scaling up [26]. Shopperly et al. [27] demonstrated that stereolithographic 3D bioprinting holds significant potential for fabricating biomimetic cartilage-like tissue, highlighting that a more biomimetic approach can be achieved by blending and stratifying methacrylated hyaluronic acid (HAMA) and methacrylated gelatin (GelMA) bioinks to replicate the zonal architecture of articular cartilage. Table 1 summarizes comparison of main 3D bioprinting techniques.

**Table 1:** Comparison of main 3D bioprinting techniques and their characteristics

Technology	Resolution	Speed	Materials	Advantages	Limitations	Refs.
Extrusion-based (pneumatic, piston, or screw-driven)	Moderate	Medium	Hydrogels, composite bioinks, thermoplastic	High versatility, supports viscous materials	Expensive setup	[140]
Inkjet 3D bioprinting	High	High	Bioinks, polymer solutions, hydrogels	High precision, efficient bioink control, low cost, high-cell viability, rapid deposition	Limited material compatibility, poor viscosity handling, limited layers, and limited to low-viscosity materials	[4, 141–143]
Laser-assisted 3D bioprinting	Very high	Slow	Bioinks with photosensitive polymers	High accuracy, good cell viability, nozzle-free	Expensive, slower throughput, potential UV damage to cells	[141, 144]
Stereolithography	Very high	Slow	Photosensitive resins, hydrogels	Excellent resolution and accuracy, Scalable	Toxic resins and limited material options, expensive, required photo-initiators	[144, 145]
Sacrificial writing	High	Moderate	Gelatin, collagen	Creates precise hollow channels, versatile	Limited scalability, removal of core material	[146]
Dynamic interface printing	Medium	Very fast	Resin bubbles, light-sensitive materials	Fast fabrication, soft tissue replication	Limited to small-scale structures, precision issue	[146]
Co-SWIFT	High	Moderate	Collagen	Mimics native vascular architecture, high viability	Complex setup, difficulty in capillary formation	[146]
Magnetic 3D bioprinting	High	High	Pre-magnetized bioinks	No nozzle clogging, precise spatial control	Limited clinical research	[98]

Although fused deposition modeling (FDM) is one of the most widely used AM methods, its role in 3D bioprinting is more limited. FDM primarily works by extruding thermoplastic filaments through a heated nozzle, making it highly suitable for fabricating acellular scaffolds and support structures [28]. However, due to the high processing temperatures, FDM is generally not employed for direct cell-laden printing, which differentiates it from true 3D bioprinting techniques, such as extrusion, inkjet, laser-assisted, or stereolithography [29]. Instead, FDM is often applied in hybrid approaches, where polymeric scaffolds fabricated via FDM are subsequently seeded with cells or combined with hydrogel-based 3D bioprinting. Its advantages include affordability, material versatility (e.g., PCL and PLA), and the ability to generate mechanically robust structures for bone and cartilage regeneration [30]. Nevertheless, the lack of cell compatibility during the printing stage and limited resolution for vascularized constructs restrict its direct use in 3D bioprinting. Therefore, FDM is better considered as a complementary AM tool rather than a core 3D bioprinting technique [31].

Sousa et al. [32] highlighted that 3D printing, as a widely used additive manufacturing technique, enables the fabrication of biostructures for tissue engineering applications, including bone, orthopedic tissues, and organs, emphasizing the critical role of scaffold manufacturing techniques and material selection in determining the structural, mechanical, and biological performance of implanted biomaterials in bone tissue engineering (BTE). Comparison of 3D bioprinting technologies, and applications are listed in Table 1.

## Advanced biomaterials

The success of 3D bioprinting depends on bioinks that support cell growth and tissue formation. Advanced hydrogels, decellularized extracellular matrices (ECM), and self-healing, and -assembling materials have emerged as pivotal in mimicking native tissues’ properties. These innovations enhance the fidelity and functionality of 3D bioprinted tissues, paving the way for creating more complex organ systems [33].

### Hydrogels as bioinks

Hydrogels remain the cornerstone of 3D bioprinting because of their biocompatibility, high water content, and ability to encapsulate cells. However, their successful application relies heavily on tailoring their physicochemical properties to balance printability, mechanical stability, and biological performance [34]. Recent reviews emphasize that strategies such as chemical crosslinking, rheological tuning, and incorporation of functional biomolecules are essential for improving cell viability and tissue-specific outcomes [35, 36].

Hydrogels remain the cornerstone of 3D bioprinting because of their biocompatibility, high water content, and ability to encapsulate cells [37]. However, their successful application relies heavily on tailoring their physicochemical properties to balance printability, mechanical stability, and biological performance [38]. Recent reviews emphasize that strategies such as chemical crosslinking, rheological among polysaccharide-based hydrogels, hyaluronic acid (HA), alginate, and agarose are widely studied [39]. Native HA supports cell proliferation and cartilage homeostasis but suffers from weak mechanics; crosslinking with aldehyde or thiol groups enhances its load-bearing capacity [40]. Alginate is inexpensive and biocompatible but has poor cell adhesion; functionalization with RGD peptides or ECM-derived motifs improves bioactivity, making it attractive for cardiovascular and bone regeneration [41].

Agarose provides mechanical stability but limited cell proliferation; blending with gelatin or alginate enhances its biological functionality, particularly for cartilage and vascular models [42]. Together, these polysaccharide hydrogels offer tunable biochemical and mechanical profiles but generally require modification for optimal performance [43]. In contrast, protein-based hydrogels such as collagen and gelatin derivatives provide intrinsic bioactivity [44, 45]. Collagen supports adhesion and differentiation but has weak mechanical strength; photo-crosslinking improves stability and transparency, making it suitable for corneal tissue engineering [46]. Gelatin, derived from collagen, is often modified with GelMA, enabling photo-crosslinking and greater mechanical control. Adjusting the degree of methacrylation or blending with microspheres expands GelMA’s use in vascular, bone, and liver tissue models, highlighting its versatility [47]. For load-bearing applications, silk fibroin is particularly promising. It provides outstanding mechanical strength and biodegradability, but low viscosity limits printability. Chemical modifications such as methacrylation enable photo-crosslinking and improved rheological properties, expanding its utility in bone and musculoskeletal tissue engineering [48].

Moreover, SF-based smart hydrogels have been explored for their stimuli-responsive behavior, tunable mechanical properties, and suitability for drug delivery and regenerative medicine applications, providing versatile platforms for tissue engineering [49]. Overall, these examples demonstrate that the performance of natural hydrogels in 3D bioprinting is highly dependent on modification strategies that tailor printability, degradation, and bioactivity to meet application-specific requirements [50].

Despite their promise, most hydrogels exhibit intrinsic challenges including mechanical weakness, limited stability under physiological stress, and reduced cell viability due to shear forces during extrusion [51]. Current research addresses these limitations through (i) optimizing crosslinking density, (ii) viscosity and rheological control, and (iii) functionalization with bioactive peptides or nanomaterials [35, 52]. Applications of hydrogel-based bioinks are listed in Table 2. Notably, DNA-based biomolecules have recently emerged as programmable bioinks with tunable stiffness, degradation, and cell-instructive properties, representing a new class of “smart” hydrogel systems [53].

**Table 2:** Applications of different bioink types, including their properties, specific uses, and limitations

Bioink type	Properties	Applications	Limitations	Refs.
Decellularized ECM	Mimics native tissue, Nutrient-rich	Organ scaffolds, islet encapsulation, vascularized tissues, urethra, bladder tissue regeneration, orbital implants, corneal layers, hepatic tissue scaffolds	High cost, Variability, Batch variability, Risk of contamination from animal sources	[2, 121, 142, 143, 147, 148]
Collagen	Bioactive, flexible, biocompatible, promotes keratocyte adhesion, transparent, support cell adhesion	Skin, cartilage, vascular structures, heart tissue, epithelial, and stromal tissue, corneal tissue engineering	Degradation without crosslinking, Low mechanical strength, High cost, Limited mechanical properties	[145, 146, 148, 149]
Gelatin	Biocompatible, biodegradable, sacrificial material, easy removal	Cartilage engineering, hollow channels, perfusion studies	Low mechanical strength, Not suitable for structural support	[4, 146]
Chitosan	Anti-bacterial, biodegradable, biocompatible, supports wound healing	Neural and bone tissues, skin and cartilage scaffolds, alveolar scaffolds, lung tissue models	Low stability without crosslinking, Slow gelation, Poor mechanical strength	[4, 140, 150]
PLA	Durable, excellent mechanical properties, tunable properties	Hard tissue engineering, urological scaffolds, facial prosthetics, load-bearing liver scaffolds	High temperature excludes cells, Biocompatibility challenges	[4, 98, 121, 147]
PCL	Strong supports structural integrity	Load-bearing lung scaffolds	Limited cell compatibility	[150]
Gelatin methacrylate	Biocompatible, photopolymerizable, high-cell viability, transparent	Heart patches, cartilage, corneal stroma regeneration	Requires UV or visible Light for crosslinking, Limited mechanical strength	[144, 149]
Synthetic PEG	Tunable mechanical properties	Bone, cartilage models, islet scaffolds	Low bioactivity unless functionalized	[142, 144]
Gelatin–alginate	Biocompatible, supports cell adhesion, tunable viscosity	Pancreatic islets, vascular scaffolds	Limited mechanical strength, Requires crosslinking agents	[142, 143]
PEG–DA-based bioinks	Photo curable, high resolution	Capillary structures	Requires UV exposure, Potential cell damage	[143]
Stem cell-based bioinks	Enables differentiation into multiple cell types	Personalized implant, disease models	Limited scalability, cost intensive	[85]
Sodium alginate	Biodegradable, hydrophilic	Structural scaffolds for corneal tissue	Poor mechanical properties	[149]
Agarose	Thermosensitive, allows sacrificial patterning	Vascularized tissue models	Non-adhesive, limited cell support	[140]
Hyaluronic acid	Highly viscous, supports cell adhesion	Soft tissue engineering	Rapid degradation, Requires modification	[140]
Nanocellulose	High mechanical strength, biodegradable	Bone scaffolds, vascular models	Complex processing requirements	[151]

A critical challenge in hydrogel bioinks is supporting vascularization of thick or highly cellular constructs. Recent experimental strategies have attempted to tackle this through sacrificial bioink printing—where temporary channels (e.g., using gelatin and Pluronic F127) are embedded and later removed to create perfusable microvessels [46, 54, 55]. Another promising approach utilizes microfluidics-integrated 3D bioprinting systems to pattern endothelial cells within hydrogel matrices, fostering lumen formation and angiogenic sprouting under controlled flow conditions [56]. Advanced reviews have also highlighted embedded sacrificial printing for hierarchical vascular networks in complex tissues [57, 58]. These vascularization strategies, while still in preclinical stages, represent crucial early steps toward overcoming perfusion limitations in 3D bioprinted tissues.

### Advanced hydrogels

Advanced hydrogels are distinguished from conventional hydrogels by their ability not only to provide a supportive environment for cells, but also to actively regulate biological functions or respond to external cues. While standard hydrogels are often inert scaffolds requiring additional modification, advanced hydrogels incorporate functional motifs or dynamic properties that enable more sophisticated tissue engineering applications [59].

One important class of advanced hydrogels incorporates cell-adhesive peptides, such as RGD sequences, which enhance integrin-mediated adhesion, signaling, and tissue-specific differentiation [59]. Such biofunctionalization improves outcomes in wound healing and regenerative contexts. Another strategy involves integration of carbon nanomaterials (CNMs), such as graphene oxide or carbon nanotubes, which reinforce hydrogel mechanics and impart electrical conductivity—properties that are particularly valuable for cardiac and neural tissue models [60].

In addition, stimuli-responsive or “smart” hydrogels have emerged, capable of altering their properties in response to pH, temperature, or light [59, 61]. This adaptability supports applications in controlled drug delivery, dynamic tissue remodeling, and 4D bioprinting [62]. Closely related are self-healing hydrogels, which exploit reversible covalent or supramolecular bonds to restore their integrity after deformation, thereby enhancing durability in load-bearing constructs [63]. Finally, ECM-mimicking hydrogels derived from decellularized extracellular matrices replicate the biochemical and structural features of native tissues, offering highly physiologically relevant microenvironments for embedded cells [64]. By closely reproducing the native milieu, these bioinks improve cell viability, differentiation, and long-term function [65].

**Fig. 2:**
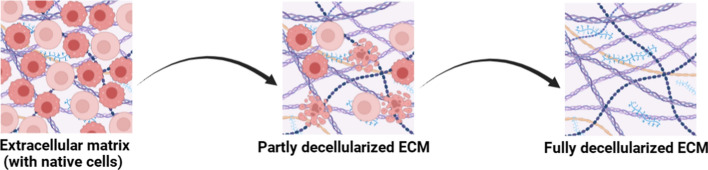
Step-by-step decellularization of the extracellular matrix: cells are removed from tissues while preserving the matrix's biochemical and structural integrity

By incorporating such functionalities, advanced hydrogels overcome many limitations of traditional bioinks and provide tailored solutions for diverse applications ranging from regenerative medicine to disease modeling. Nevertheless, challenges remain in standardizing fabrication methods, ensuring reproducibility across laboratories, and scaling production for clinical use [66]. Among advanced hydrogels, decellularized extracellular matrix (dECM)-based bioinks represent one of the most biologically relevant classes. Because they retain native biochemical cues and structural complexity, they serve as tissue-specific hydrogels with superior physiological fidelity. dECM is obtained by removing cells from tissues or organs (Fig. 2) while preserving the biochemical and structural integrity of the matrix. This process produces dECM that provides a highly biomimetic environment for cells, enabling the creation of organ-like structures with realistic mechanical and biological properties. These dECM-based bioinks promote cell attachment, growth, and differentiation, making them ideal for organ fabrication [67].

The decellularization process involves physical, chemical, and enzymatic techniques to remove cellular components while retaining the extracellular matrix. The choice of method depends on tissue type, density, and ECM composition, which influence regenerative potential and the properties of the resulting scaffold. Post-decellularization, tissues act as natural biomaterials providing structural and biochemical support, facilitating cell communication and tissue engineering applications [68]. Effective decellularization and validation protocols are crucial for ensuring reproducibility and functionality of dECM bioinks. These bioinks are increasingly used to formulate tissue-specific constructs that mimic native microenvironments, supporting applications in regenerative medicine and biofabrication. The field continues to explore standardized procedures and characterization methods to optimize the safety, efficacy, and clinical translation of dECM-based bioinks.

dECM has broad applications in tissue engineering due to its ability to retain essential mechanical properties and bioactive factors. In bone and cartilage regeneration, dECM derived from these tissues provides the mechanical properties and growth factors necessary for osteogenic and chondrogenic differentiation, making it ideal for scaffolds used in orthopedic applications [69].

For vascularized tissues, decellularized vascular structures are repopulated with endothelial and smooth muscle cells to create bioengineered blood vessels, which are crucial for enabling tissue perfusion in large-scale engineered organs. In soft tissue repair, dECM scaffolds derived from adipose tissue and skin are employed in wound healing, reconstructive surgery, and cosmetic procedures, leveraging their flexibility, and bioactivity [70]. Furthermore, in organ engineering, decellularized whole organs, such as the liver, heart, and kidney, serve as frameworks for recellularization with patient-specific cells, offering significant advancements in organ transplantation possibilities. Shin et al. [71] developed hydrogel bioinks composed of partially digested porcine cardiac decellularized extracellular matrix (cdECM), laponite–XLG nanoclay, and poly(ethylene glycol)–diacrylate (PEG–DA), combining extrudability, shape fidelity, rapid cross-linking, and cytocompatibility, with over 97% viability of human cardiac fibroblasts and over 94% viability of human induced pluripotent stem cell (iPSC)-derived cardiomyocytes after 7 days.

dECM offers several advantages that make it highly suitable for biomedical applications. Its biocompatibility is a key feature, as the native ECM composition supports cell adhesion, proliferation, and differentiation. In addition, dECM scaffolds can be customized to specific tissues, providing targeted growth factors tailored to various applications. The decellularization process also significantly reduces immunogenicity, minimizing immune responses in recipients and enhancing the safety and efficacy of dECM-based therapies [70].

Despite its potential, decellularized extracellular matrix dECM faces several challenges that limit its effectiveness in regenerative medicine and tissue engineering. Retention of bioactivity is a significant issue, as prolonged or harsh decellularization processes can damage essential ECM components, such as growth factors and proteins, reducing the scaffold's effectiveness. Mechanical integrity is another concern, as dECM hydrogels often lack sufficient mechanical strength for load-bearing applications, requiring reinforcement with synthetic materials [72]. In addition, the lack of standardization in decellularization techniques leads to variability in scaffold properties, impacting reproducibility in both research, and clinical applications.

To address these challenges, strategies such as combining dECM with synthetic polymers or nanomaterials to enhance mechanical properties and bioactivity are being explored. 3D Bioprinting techniques are also being investigated to incorporate dECM into customized tissue constructs for patient-specific therapies [73]. Furthermore, advancing decellularization protocols to better preserve ECM structure while enabling scalable production is essential for realizing the transformative potential of dECM in regenerative medicine.

### Self-healing biomaterials

Self-healing hydrogels are innovative biomaterials capable of autonomous repair after mechanical damage, which extends their durability and functionality. These hydrogels mimic natural tissue properties, making them suitable for biomedical applications, including tissue engineering, wound healing, and drug delivery [74].

Self-healing hydrogels utilize various mechanisms to restore their structural integrity and functionality. Covalent interactions involve dynamic covalent bonds, such as Schiff bases, disulfide bonds, and boronate ester linkages, which provide strong and reversible self-repair capabilities [75]. These mechanisms are often stimulus-responsive, triggered by environmental changes, such as pH, light, or temperature. Non-covalent interactions, including hydrogen bonding, ionic bonding, and host–guest interactions, such as β-cyclodextrin–adamantane complexes, enable fast healing while maintaining hydrogel flexibility, making them ideal for soft tissue applications. Hybrid approaches combine both covalent and non-covalent interactions, offering enhanced mechanical strength and adaptability to meet the demands of complex physiological environments [76].

Self-healing biomaterials face several challenges that must be addressed to enhance their effectiveness in biomedical applications. One key issue is balancing strength and healing efficiency, as improving mechanical properties often compromises the self-healing ability, which is particularly problematic for load-bearing applications. Cytotoxicity of components, such as certain crosslinkers and synthetic polymers, is another concern, requiring careful optimization for biocompatibility. In addition, ensuring the long-term stability of hydrogels under physiological conditions and during repeated healing cycles is crucial for their practical use [74].

Advancements in multi-stimuli-responsive hydrogels, such as those sensitive to pH and temperature, are expected to expand their clinical applications. Moreover, integrating nanomaterials such as graphene or carbon nanotubes to enhance conductivity and mechanical properties holds promise for applications in neural, and cardiac tissue engineering [77]. These developments underscore the potential of self-healing hydrogels as next-generation materials for diverse biomedical fields.

### Self-assembling materials

Emerging bioinks include self-assembling peptides that form nanostructures mimicking native tissue at the molecular level. These materials are particularly promising for creating highly detailed tissues, such as capillaries or alveoli. Self-assembling peptide hydrogels are versatile biomaterials that form stable, water-retaining structures through non-covalent interactions, such as hydrogen bonding, hydrophobic effects, and π–π stacking. These hydrogels mimic the ECM, and are widely used in tissue engineering, drug delivery, and regenerative medicine due to their biocompatibility, biodegradability, and ability to encapsulate bioactive molecules [78].

Peptide hydrogels are formed through spontaneous assembly of amino acids into secondary structures such as β-sheets or α-helices under physiological conditions [79]. Stimuli-responsive hydrogels change properties based on pH, temperature, or ionic strength, enhancing their versatility in biomedical applications.

Mechanical stability and gelation conditions can be adjusted by modifying peptide sequences or adding functional groups like RGD, which improves cell adhesion and proliferation [78].

Self-assembling materials, particularly hydrogels, face several challenges that limit their broader application in bioengineering. One major issue is stability and scalability, as these hydrogels may lack the mechanical robustness required for load-bearing applications, restricting their use in certain areas. Another challenge is degradation control, as ensuring consistent degradation rates in vivo that align with tissue regeneration timelines remains difficult. Despite these hurdles, self-assembling hydrogels represent cutting-edge materials with significant potential for personalized therapies and advanced regenerative solutions in the field of bioengineering [80] (Table 2).

## Key application in medicine

“Before discussing tissue-specific applications, it is important to highlight that certain advanced biomaterials—particularly self-healing and self-assembling hydrogels—play broad and cross-cutting roles across multiple bioprinting contexts.”

Self-healing hydrogels have a wide range of applications in biomedicine, offering innovative solutions for various challenges. In tissue engineering, they are used to construct load-bearing scaffolds capable of autonomously healing micro-fractures, while hydrogels enhanced with RGD peptides promote cell adhesion and growth, supporting bone and cartilage regeneration. For wound healing, injectable hydrogels provide a protective matrix that reduces inflammation, accelerates healing, and delivers encapsulated growth factors and antimicrobial agents [81]. In drug delivery, these hydrogels enable localized and controlled therapeutic release, with their self-healing properties ensuring sustained functionality even under mechanical stress. In addition, the dynamic characteristics of self-healing hydrogels make them ideal as bioinks for 3D bioprinting, facilitating the fabrication of complex tissue structures. Daly et al. [82] developed a high-resolution 3D bioprinting approach to transfer spheroids into self-healing support hydrogels, enabling their patterning and fusion into high-cell density microtissues. As an application, they 3D bioprinted induced pluripotent stem cell-derived cardiac microtissues with controlled cardiomyocyte and fibroblast ratios to replicate the structural and functional features of scarred cardiac tissue following myocardial infarction.

Self-assembling materials, particularly hydrogels, have significant applications in various biomedical fields due to their ability to mimic natural structures and respond to environmental stimuli. In drug delivery, hydrogels enable controlled and localized release by encapsulating small molecules and biologics. For example, NapGFFY hydrogels have demonstrated excellent drug loading capacity and stimuli-responsive release, making them ideal for chemotherapy applications. In tissue engineering, these hydrogels mimic the ECM, supporting cell growth and tissue regeneration, which makes them suitable for engineering bone, cartilage, and neural tissues. RGD-modified hydrogels specifically enhance interactions with integrins, promoting cell anchorage during tissue formation [83]. In cancer therapy, functionalized peptide hydrogels can target tumors by delivering anticancer drugs or sequestering growth factors like vascular endothelial growth factor (VEGF), thereby inhibiting angiogenesis and reducing tumor invasion and metastasis [78]. Bakht et al. [84] combined the controlled self-assembly of plant-derived cellulose nanocrystals (CNC) with 3D bioprinting in suspension baths to biofabricate microphysiological systems embedded in an ECM-mimetic fibrillar support material. The CNC fluid gel developed enables high-resolution 3D bioprinting of 3D constructs with arbitrary geometries and minimal restrictions on bioink selection.

3D Bioprinted tissues are already being tested in regenerative medicine. For example, 3D bioprinted skin has shown promise in treating burn victims, while 3D bioprinted cartilage and bone tissues are being explored for orthopedic applications. However, the most exciting applications are in organ regeneration. Scientists are working toward printing liver and kidney tissues, which could eventually lead to the development of full transplantable organs. In addition, patient-specific tumor models are paving the way for personalized drug testing, allowing doctors to tailor treatments based on how the printed tissues respond to drugs [85]. Applications of 3D bioprinting in medicine are listed in Table 3.

**Table 3:** Applications, challenges, and proposed solutions in 3D bioprinting

Application area or Tissue type	Current status	Main challenges	Proposed or emerging solutions	Refs.
Skin regeneration	Preclinical and clinical trials	Achieving vascularization, multilayer complexity	Use of vascular growth factors, co-printing endothelial cells, hydrogel gradients	[2, 4, 142]
Liver models	Experimental, Drug testing, disease modeling	Functional vasculature, metabolic activity	Incorporation of hepatocytes and endothelial co-cultures, perfused chip systems	[121, 144, 152]
Bone and cartilage repair	Experimental, Early preclinical	Mechanical strength, integration with host tissue	Hybrid bioinks (e.g., PCL/hydrogel), Crosslinking enhancement	[141, 146]
Cardiac and vascular tissues	Preclinical models, Functional prototypes	Synchronization of beating, vascular integration	Multi-nozzle printing, electrical stimulation, biomimetic vascular scaffolds	[144, 146, 152]
Neural tissue engineering	Early stage research	Functional integration and innervation	Co-culture with glial cells, neural growth factor incorporation	[4, 85]
Lung and respiratory models	In vitro research and infection testing	Replicating alveolar–capillary structures, vascularization	Sacrificial bioinks, microfluidic-based printing, perfusable constructs	[144, 146, 150]
Pancreatic and diabetes models	Preclinical	Maintaining β-cell viability and function	Use of ECM-mimetic hydrogels, vascularized islet organoids	[142, 146]
Corneal and ocular tissue engineering	Preclinical and experimental	Optical transparency, mechanical strength	GelMA or collagen-based bioinks, gradient refractive bioinks	[148, 149]
Kidney and urinary tissue models	Drug screening, Early preclinical	Complex filtration and multicellular organization	Organ-on-chip integration, bioprinted nephron units	[85, 147]
Soft tissue and muscle repair	Limited clinical use	Structural stability and mechanical compliance	Use of elastomeric hydrogels, bioelectronic Stimulation	[98, 146]
Tumor and disease modeling	Preclinical, Drug testing	Standardization and reproducibility	AI-guided bioprinting, Integrated sensors for real-time monitoring	[85, 142]
Vascularization (cross-cutting issue)	Key across all applications	Nutrient/oxygen diffusion limits	Growth factor delivery, Sacrificial channels; bioreactors	[142, 143, 152]
Immune rejection	Translational barrier	Host compatibility	Use of autologous or iPSC-derived cells, Immunomodulatory coatings	[2, 142]
Scalability and cost	Technical challenge	Low throughput, Expensive systems	Multi-head printers, Low-cost bioinks, Open-source printing platforms	[2, 146]
Printability and structural stability	Common technical limitation	Layer collapse, Inconsistent deposition	Rheological optimization, Crosslinking control	[140, 143]
Regulatory and clinical translation	Key barrier to adoption	Lack of standardized protocols	International standards (ISO/FDA), Quality control frameworks	[85, 146]

### Regenerative medicine

**Fig. 3:**
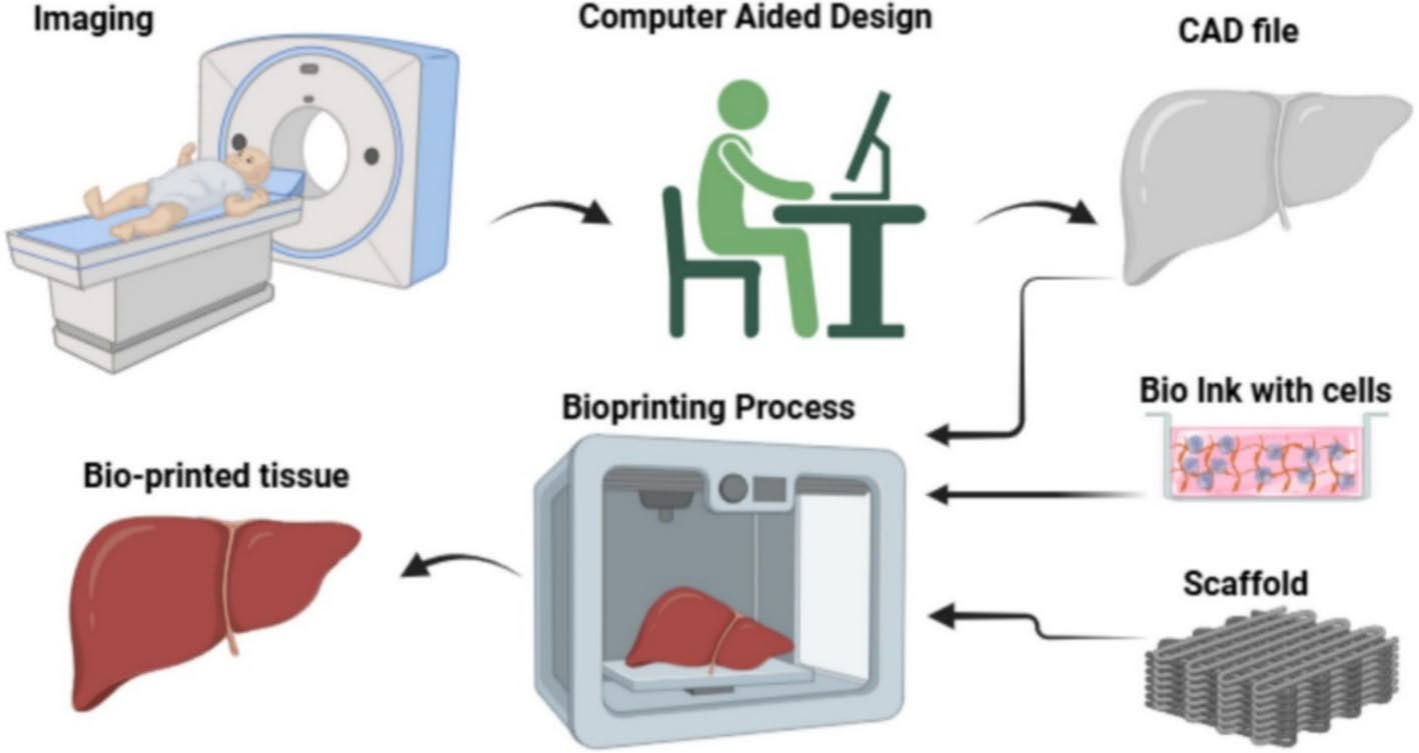
Schematic representation of the 3D bioprinting process in regenerative medicine. The illustration highlights the fabrication steps: imaging, computer-aided design, 3D bioprinting process with the aid of bioinks and bioprinter

3D bioprinting in regenerative medicine (Fig. 3) is an innovative approach that combines bioengineering and medicine to create functional tissues and organs. Using bioinks composed of living cells and biomaterials, structures are built layer by layer. As shown in Fig. 3, this technology supports applications, such as tissue regeneration, organ transplantation, and drug-testing models [86, 87]. It allows for patient-specific solutions, improving outcomes in clinical settings. Despite challenges, such as ensuring vascularization and mechanical integrity, 3D bioprinting holds significant potential for addressing organ shortages, and advancing personalized medicine, although these goals remain major challenges for clinical translation.

Samandari et al. [88] highlighted the limitations of conventional in vitro 3D bioprinting for fabricating irregularly shaped scaffolds and introduced in situ 3D bioprinting as an emerging strategy for direct bioink deposition in vivo, discussing its advantages, challenges, and potential future advancements. Nanmo et al. [89] proposed a scalable 3D bioprinting approach for hair-inductive tissue grafts by printing collagen microgels with mesenchymal and epithelial cells, enabling enriched cell density and efficient hair follicle regeneration. Using suture guides, they improved hair-shaft sprouting through controlled microgel orientation during transplantation.

### Personalized medicine

3D Bioprinted patient-specific tissues are being used in drug testing, particularly in cancer research. Tumor models printed using the patient’s own cells can be used to test various drug treatments and predict the best course of action, reducing the reliance on animal testing. In the future, these tissues could also be used to simulate complex multidisease models, offering a more accurate prediction of how drugs will affect human bodies [90]. This personalized approach is expected to drastically improve the development of tailored medical treatments, and precision medicine.

For example, 3D bioprinting enables the creation of patient-specific cancer models, replicating the tumor microenvironment and interactions between cancer cells, ECM components, fibroblasts, and immune cells. Personalized 3D bioprinted models also allow researchers to test the effectiveness of therapeutic approaches and drug regimens on an individual’s tumor before clinical application, improving treatment precision and outcomes. In addition, 3D bioprinted tumor models can be preserved as biobanks, helping to understand treatment responses across patient cohorts and contributing to the development of broader therapeutic strategies. Furthermore, 3D bioprinting supports the study of cancer stem cells (CSCs), which are often implicated in treatment resistance and relapse. By mimicking the native tumor environment, 3D bioprinted constructs enable the enrichment and study of CSCs for the development of targeted therapies [91]. Kim et al. [92] developed a vascularized organoid model (VOM) comprising patient-derived gastric cancer organoids (PDOs), perfusable endothelium, and stomach dECM, enabling accurate prediction of clinical responses to VEGFR2-targeted therapy based on PDO molecular subtypes.

Beyond oncology, personalized 3D bioprinting has been applied to generate patient-specific cardiac, hepatic, and neural tissue models for assessing drug-induced toxicity and organ-specific side effects in preclinical studies. For instance, patient-derived cardiac organoids have been used to evaluate cardiotoxicity of chemotherapeutic agents in models of inherited cardiac disorders [93], while liver organoids derived from iPSC-hepatocytes allow prediction of hepatotoxic responses before clinical trials [94]. These models provide predictive value that conventional 2D cultures or animal models cannot offer, particularly for cardiotoxicity and hepatotoxicity, which remain leading causes of late-stage drug failure.

Integration with patient-specific iPSCs further enables the creation of autologous constructs that capture individual genetic backgrounds, enhancing the relevance of these models for precision medicine. Despite these advances, several challenges hinder clinical translation, including reproducibility across laboratories, scalability for large patient cohorts, and regulatory acceptance as valid alternatives to animal testing. Standardization of bioink formulations and printing protocols, along with robust validation against clinical outcomes, will be essential for their widespread adoption. Nevertheless, among the spectrum of 3D bioprinting applications, patient-specific models are among the most promising in terms of near-term impact, offering immediate utility for drug screening, treatment optimization, and reduction of animal testing.

### Surgical applications

3D bioprinting is advancing surgical applications by enabling the creation of patient-specific implants and tissues, supporting more precise and personalized treatments [95]. Customized implants, such as bone grafts, cartilage, and tissue constructs, are tailored to fit the anatomical needs of individual patients, improving the precision of surgeries, reducing complications, and enhancing healing.

For patients with severe burns or chronic wounds, 3D bioprinted skin constructs, made from bioinks, such as GelMA and collagen, offer an innovative solution, promoting better integration and faster healing compared to traditional methods [96]. In addition, 3D bioprinting has been used to create custom ear prosthetics for patients with congenital or traumatic ear deformities, utilizing DICOM images to model the ear and print it with materials that mimic the mechanical properties of ear cartilage [97, 98].

In situ 3D bioprinting, where tissue is 3D bioprinted directly in the operating room, represents a major advancement, enabling tissue regeneration on-site and reducing the need for additional surgeries [99]. Furthermore, innovations in 3D printing have improved breast augmentation techniques, offering more effective methods for both synthetic implants and autologous fat transplantation [100].

These applications in 3D bioprinting are transforming the field of surgery by providing more accurate, personalized treatments, and reducing the complexity of many surgical procedures. However, challenges such as high costs, the need for specialized expertise, and technical barriers to creating fully functional tissues with vascularity remain [98]. Thai et al. [99] introduced the multifunctional F3DB in situ bioprinter, featuring a soft printing head on a flexible robotic arm for precise multi-layered biomaterial delivery. They demonstrated its capability for endoscopic surgery on porcine tissue, highlighting its potential for advanced in situ 3D bioprinting applications.

### Wound healing

**Fig. 4:**
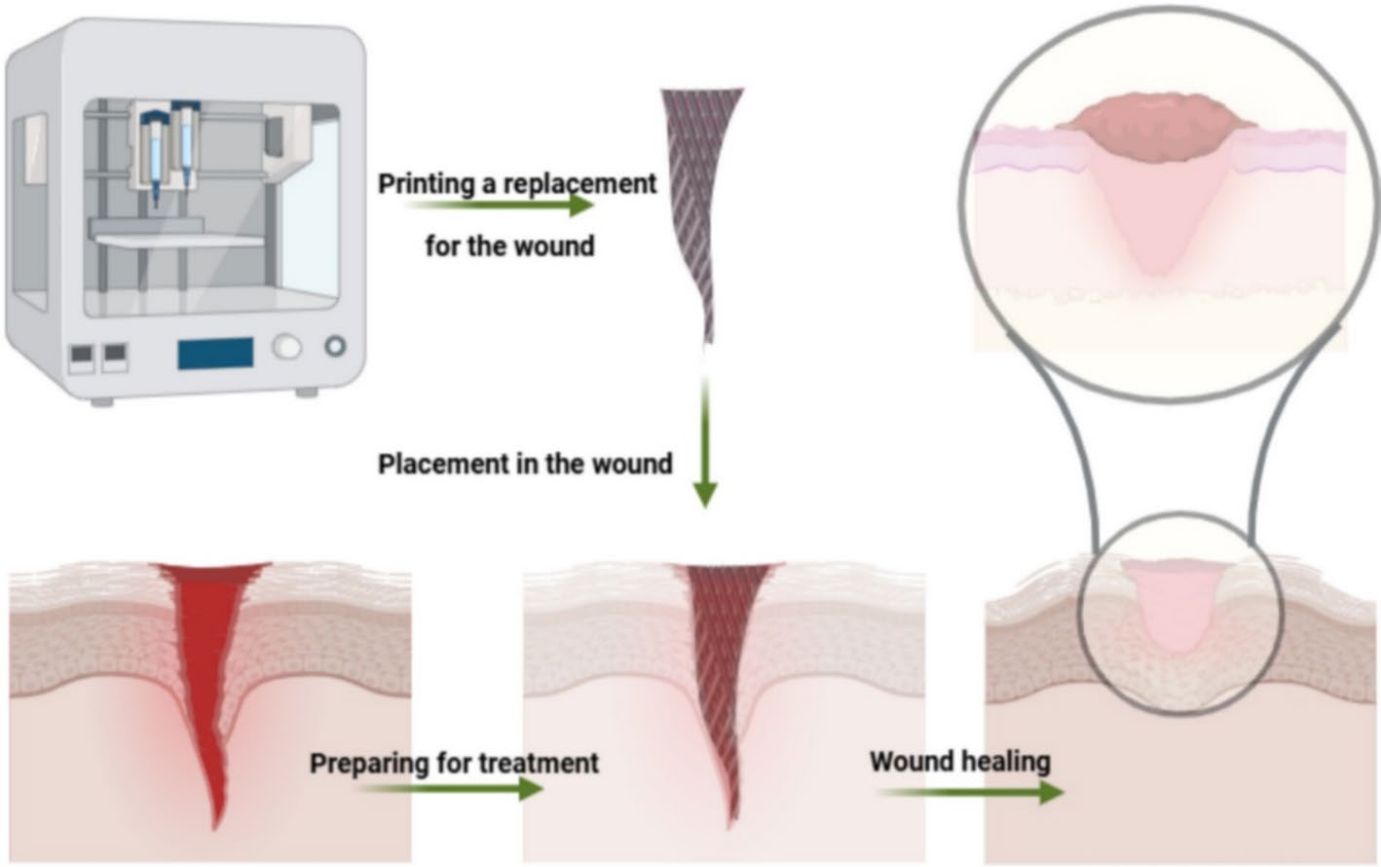
Application of 3D bioprinting in wound healing. The figure illustrates how 3D bioprinted constructs, designed with biocompatible materials and cells, provide personalized solutions for complex wounds, aiming to accelerate healing and enhance patient outcomes

3D bioprinting is advancing the field of wound healing by providing highly tailored, efficient, and biocompatible solutions for complex wounds [101]. This innovative approach, illustrated in Fig. 4, is changing how medical professionals treat injuries, with the potential to reduce healing time and improve patient outcomes, though further validation in clinical trials is needed.

One of the key developments is the creation of customized scaffolds and wound dressings. Through 3D bioprinting, personalized wound care solutions are designed to match the unique anatomical and healing needs of each patient. These tailored structures promote cell adhesion and tissue regeneration, which are critical for healing complex or chronic wounds [102]. In addition, bioinks containing living cells, growth factors, and biomaterials are being developed to print wound healing constructs that closely mimic natural tissue structures. These bioinks enhance the biocompatibility of printed scaffolds, providing an environment that supports faster tissue regeneration [103].

Furthermore, advanced 3D bioprinting techniques have led to the creation of multifunctional wound care devices, such as antimicrobial dressings, which not only support tissue growth but also prevent infection [104]. These multifunctional devices are particularly beneficial for managing chronic wounds that are prone to complications like infections. Ding et al. [105] developed an MoS₂-accelerated gelling hydrogel scaffold for in situ 3D bioprinting, promoting chronic diabetic wound healing through antioxidant and photothermal anti-bacterial properties. The printed scaffold facilitated faster wound closure, reduced oxidative stress, and eliminated bacterial infection, demonstrating its potential for chronic wound management.

### Disease modeling

**Fig. 5:**
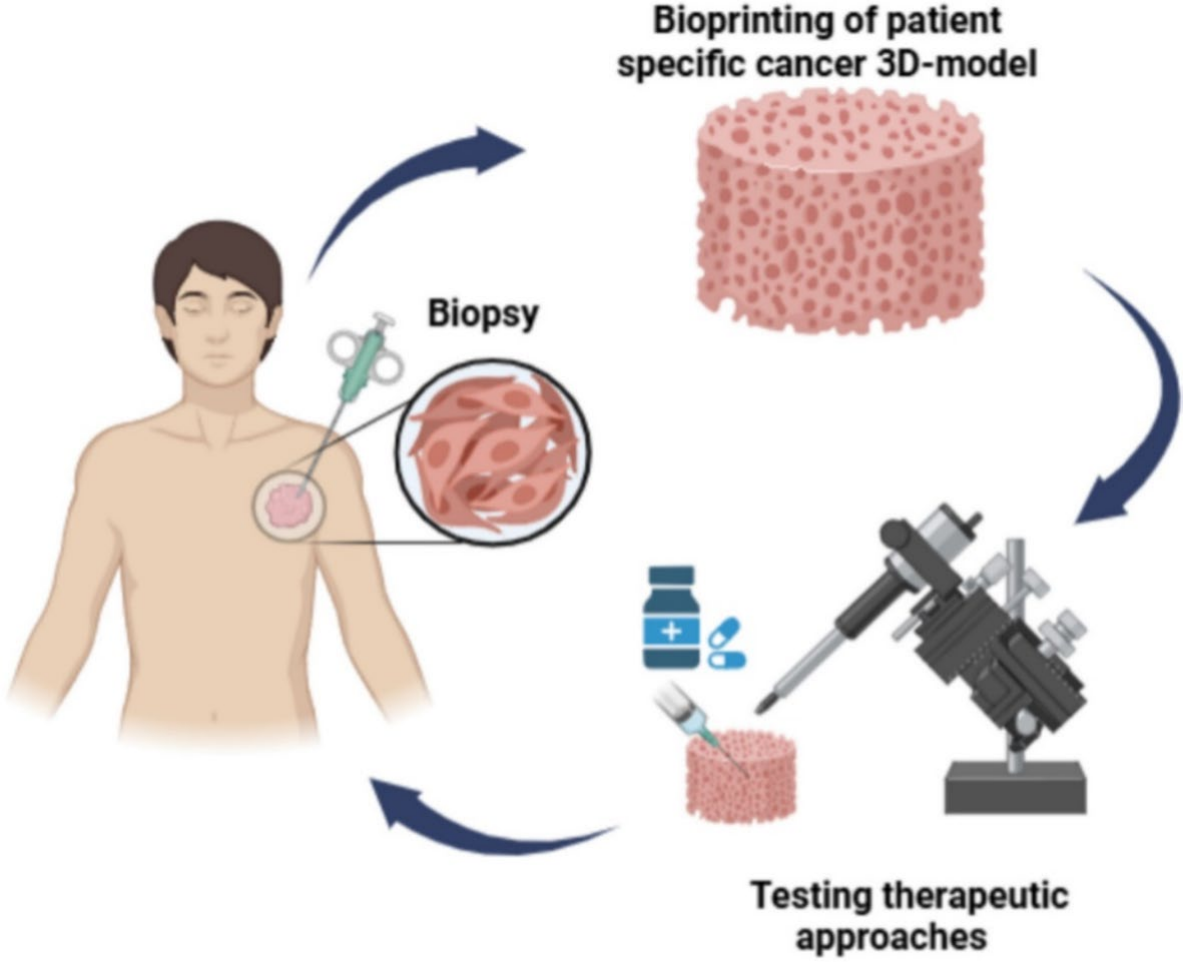
Role of 3D bioprinting in disease modeling. The figure illustrates how 3D bioprinted tissues, including skin and iPSC-derived neural constructs, replicate disease-specific conditions

Disease modeling with 3D bioprinting plays a crucial role in advancing biomedical research by enabling the creation of more accurate and functional disease models. One key application is the creation of disease-specific models. 3D Bioprinted tissues, such as skin, can mimic disease conditions, such as skin cancer, psoriasis, and chronic wounds, allowing researchers to study disease progression, test new drugs, and assess therapeutic interventions in a controlled environment before clinical trials [106]. In addition, 3D bioprinted iPSC-derived tissues are increasingly used to model complex diseases, especially those involving neural tissues, such as Alzheimer's and Parkinson's diseases [107]. As shown in Fig. 5, differentiating iPSCs into specific cell types, such as neurons or cardiomyocytes, allows researchers to replicate disease environments in vitro, providing insights into disease mechanisms and a platform for drug testing.

Another important aspect of disease modeling is the creation of personalized disease models. Using patient-derived cells, 3D bioprinting enables the development of models that reflect specific genetic variations, helping to understand how these variations affect disease outcomes and responses to treatments, which is highly relevant for personalized medicine [108]. Moreover, 3D bioprinted models can recreate the complex microenvironment of tissues, incorporating elements such as immune cells, extracellular matrix, and blood vessels to observe interactions in a more realistic setting [109]. This approach is essential for studying disease mechanisms.

Finally, 3D bioprinted models of diseased tissues offer an excellent platform for drug efficacy testing, allowing for more accurate predictions of how drugs might perform in humans while reducing reliance on animal testing and providing a quicker, more ethical alternative [110]. Through the use of 3D bioprinting, researchers can enhance our understanding of complex diseases, improve drug development, and contribute to more effective treatments in the future [106]. Alhattab et al. [111] developed an automated 3D bioprinting process using a tetramer peptide hydrogel to fabricate a 3D bone marrow (BM) niche-like acute myeloid leukemia (AML) disease model. This model supported leukemia, endothelial, and stromal cell viability, recapitulated native cell interactions, and revealed upregulated molecular pathways linked to AML drug resistance and relapse.

Recent studies have further expanded disease modeling applications to viral and infectious diseases. For instance, 3D bioprinted lung and airway models have been used to study viral infections, such as COVID-19, allowing investigation of viral entry, replication, and host immune responses under physiologically relevant conditions. Such platforms provide opportunities for rapid antiviral drug screening and vaccine development, which are difficult to achieve using traditional models.

Despite these advances, significant limitations remain. Many current disease models lack full vascularization and immune complexity, restricting their ability to mimic systemic disease progression. Moreover, reproducibility across different laboratories and scalability for high-throughput screening are still unresolved challenges. From a translational standpoint, regulatory frameworks for accepting 3D bioprinted disease models as preclinical standards are not yet established. Mallya et al. [112] provide a comprehensive overview of strategies for constructing 3D bioprinted disease models for drug testing, highlighting design considerations, biomaterial selection, and the incorporation of tissue-specific microenvironments to enhance model fidelity.

This work also discusses regulatory concerns associated with the use of 3D bioprinted disease models, emphasizing the importance of standardized protocols and validation to enable their translation into preclinical drug testing. Addressing these challenges will be crucial for moving disease models from proof-of-concept studies toward routine use in drug development pipelines. Nevertheless, among the various biomedical applications, disease modeling is one of the most dynamic and clinically relevant, with near-term potential to complement or even replace certain animal models.

### Organ transplants and regeneration

One of the most promising applications of 3D bioprinting is in organ regeneration, with current research focusing on 3D bioprinting skin, cartilage, and simpler tissues, while liver and kidney tissues are emerging as the next major focus. 3D bioprinting is being used to produce biomimetic organs, such as kidneys, hearts, and livers, tailored to patient-specific anatomical structures [113]. These 3D bioprinted organs replicate the biological functions of natural tissues, offering a potential solution to the ongoing donor shortage.

In addition to creating functional organs, 3D bioprinting enables the fabrication of tissue scaffolds that support cell growth and differentiation, playing a vital role in the repair and regeneration of damaged tissues. These scaffolds serve as a bridge between transplantation and regenerative medicine [114]. By integrating patient-derived cells, 3D bioprinting allows the creation of immunologically compatible organs, which reduces the risk of rejection and the need for lifelong immunosuppression [115].

**Fig. 6:**
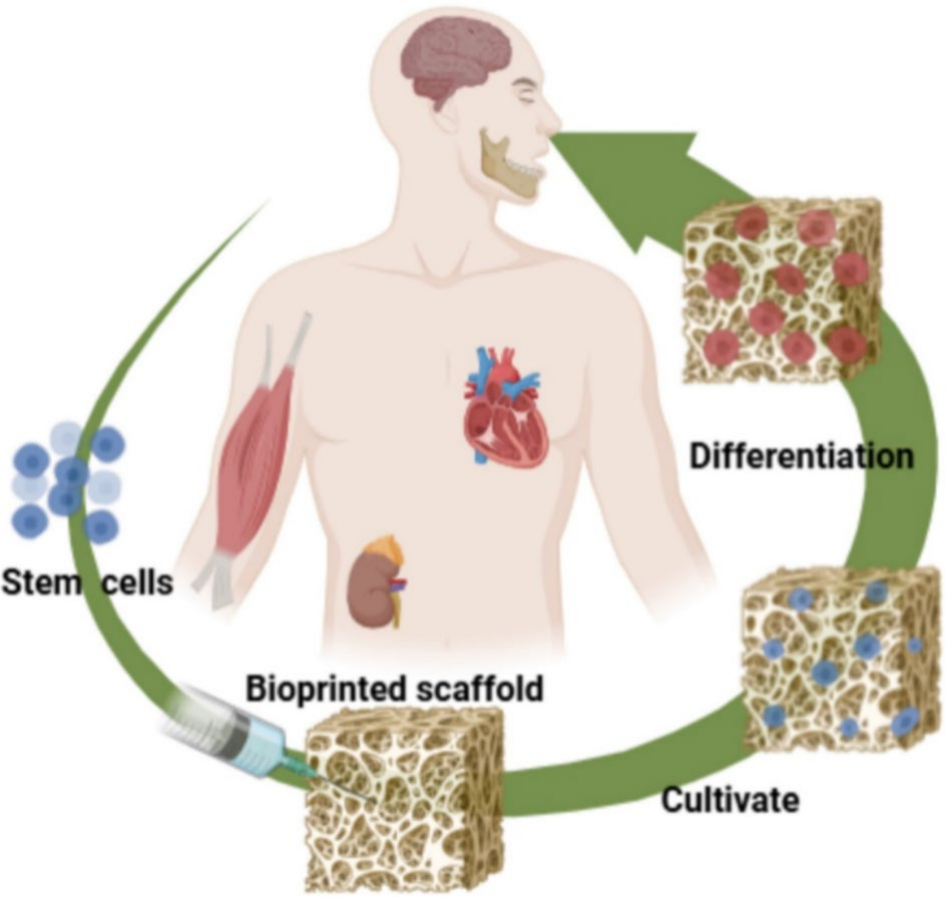
Application of 3D bioprinting in organ transplants. The figure demonstrates how combining 3D bioprinting with stem cells enables the creation of functional tissues and organoids, enhancing the biological fidelity of printed constructs and providing realistic models for transplantation and regenerative therapies

As shown in Fig. 6, combining 3D bioprinting with stem cell technology has advanced the development of functional tissues and organoids, enhancing the biological fidelity of printed constructs and providing realistic models for transplantation and disease studies [116]. Advances in printing technologies, such as extrusion-based, inkjet, and laser-assisted 3D bioprinting, provide precise control over cellular deposition and microenvironment replication, improving cell viability and structural integrity of printed tissues [117]. Goulart et al. [118] developed a novel 3D bioprinting method for functional liver grafts using iPS-derived hepatocyte-like cells, alone or with non-parenchymal cells. The resulting grafts maintained epithelial phenotype, stability, and functionality for extended periods.

While the potential of 3D bioprinting in organ regeneration is significant, challenges such as vascularization, long-term viability, and the complexity of organ functions remain to be addressed before 3D bioprinting can become a clinical standard for organ transplantation [113] (Table 3).

**3D bioprinting clinical translation challenges**

### Biological challenges

Several biological challenges must be overcome in the field of 3D bioprinting, particularly regarding cell viability and immune rejection. Maintaining high-cell viability during and after the printing process is critical, as is ensuring that the cells differentiate into the desired tissue types and perform their intended functions. This remains a significant barrier to creating fully functional 3D bioprinted tissues [119, 120].

Another challenge is immune rejection, as 3D bioprinted tissues often rely on donor-derived or non-autologous cells, which can provoke immune responses. The body may recognize these cells as foreign, leading to rejection and failure of the implant [121]. The choice of bioinks and scaffolds plays a crucial role in influencing immune reactions. Some materials may induce inflammation or an immune response, complicating the integration of the 3D bioprinted tissue with the host. To address this, developing bioinks that are both functional and immunologically inert is a critical area of research [122]. Using patient-derived cells, such as hepatocytes from iPSCs, can reduce the risk of immune rejection.

In addition, incorporating bioactive molecules into scaffolds can help modulate the immune response and enhance the integration of 3D bioprinted tissues [121]. Finally, proper vascularization is essential for the success of 3D bioprinted constructs. Inadequate vascular integration can lead to hypoxia and necrosis in the implanted tissue, triggering immune responses and impairing the functionality of the construct [123].

### Technical barriers

There are several technical barriers in the field of 3D bioprinting, particularly concerning material limitations, structural complexity, scalability, and mechanical stability. Selecting the right biocompatible and biodegradable materials for scaffolds is crucial, as these materials must support cell growth, induce regeneration, and degrade safely without triggering adverse immune reactions. Achieving the correct mechanical properties, such as strength and elasticity, is also essential to prevent failures in load-bearing applications [124, 125].

Another significant challenge is 3D bioprinting scaffolds and organoids with complex structures that mimic natural tissues, especially for bone and organ constructs. The integration of vascular networks to supply oxygen and nutrients remains a major hurdle, particularly for larger constructs, where vascularization is critical [120, 125]. In addition, scaling up production to meet clinical demands while maintaining quality presents logistical and financial challenges. The high costs associated with 3D bioprinting technologies, and materials further limit their widespread clinical use.

Finally, achieving the necessary mechanical stability for bone scaffolds is critical to support early healing without causing stress shielding, which can impair natural bone growth [125]. Many scaffolds still struggle to match the mechanical properties of natural bone, particularly in load-bearing sites.

### Regulatory and ethical considerations

As 3D bioprinting moves closer to clinical applications, it will need to navigate ethical and regulatory landscapes. Regulatory bodies, such as the FDA’s Tissue Reference Group and ISO/ASTM standards, are actively developing frameworks to classify and evaluate 3D bioprinted tissues and organs, ensuring safety, efficacy, and standardization for clinical use [126, 127]. However, the lack of universally accepted protocol for assessing long-term performance and reproducibility of 3D-printed scaffolds continues to delay clinical translation [119].

Table 3 shows key challenges in 3D bioprinting, with descriptions and proposed solutions for each challenge. Beyond regulatory challenges, ethical and legal concerns—including ownership of 3D bioprinted tissues, informed consent, and societal implications of creating functional living tissues—remain significant [128]. In addition, applications such as 3D bioprinting for drug screening offer opportunities to reduce animal testing, but also raise questions about the ethical use of human-derived materials [129]. Together, these regulatory, ethical, and technical considerations form a critical context for advancing 3D bioprinting toward safe and socially responsible clinical implementation.

Recent developments offer hope for clinical translation but also reveal significant hurdles. For instance, Inventia Life Science’s LIGŌ system has commenced a world-first clinical trial for direct, near wound-site skin 3D bioprinting using patient-derived cells to treat burn injuries, demonstrating both safety and rapid pain reduction [130]. Another notable example involves the implantation of a patient-specific 3D bioprinted ear, representing a pioneering clinical application of tissue-engineered cartilage [131]. Beyond these cases, cost and market adoption barriers remain substantial. A recent scoping review identified that only 11 clinical 3D bioprinting trials were registered globally between 2016 and 2023, underscoring the slow pace of translation and the need for standardized regulatory pathways, reimbursement frameworks, and manufacturing scalability [132].

From the regulatory standpoint, early guidance from entities such as the FDA’s Tissue Reference Group and ISO/ASTM standards bodies provides a starting point, yet clear clinical guidelines and approval protocol specific to 3D bioprinted constructs are not yet fully established [133]. Moreover, high production costs and lack of reimbursement mechanisms pose the most immediate market adoption obstacles. Addressing these barriers will be essential to facilitating the transition of 3D bioprinted products from experimental models to approved therapeutics and devices.

## Future prospects

The future of 3D bioprinting holds promising developments, particularly in areas, such as bioink customization, cell viability, and complex organ development. One of the key advancements is enhanced bioink customization through microgels, which offer adjustable properties, such as pore size, mechanical strength, and biocompatibility [134]. These features make microgels ideal for creating precise and functional tissue constructs, supporting more complex tissue engineering, and regenerative medicine applications.

In addition, microgels provide an optimal environment for cell growth, improving nutrient exchange and metabolic activity, which is expected to enhance the functionality and longevity of engineered tissues [135]. The modular and scalable nature of microgels also positions them as a leading material for constructing intricate structures, such as vascularized organs and multi-layered tissues, bridging the gap between research models and clinically viable organ replacements [136].

Emerging 3D bioprinting methods, such as microfluidic and sacrificial printing strategies, are incorporating microgels to achieve high-resolution structures and reduce fabrication time, thus improving scalability and accessibility [137]. Furthermore, microgel-based 3D bioprinting holds potential beyond medicine, with applications in drug delivery systems, cancer modeling, and the creation of biomimetic structures for research, extending its impact across biomedical science and engineering [138].

**Fig. 7:**
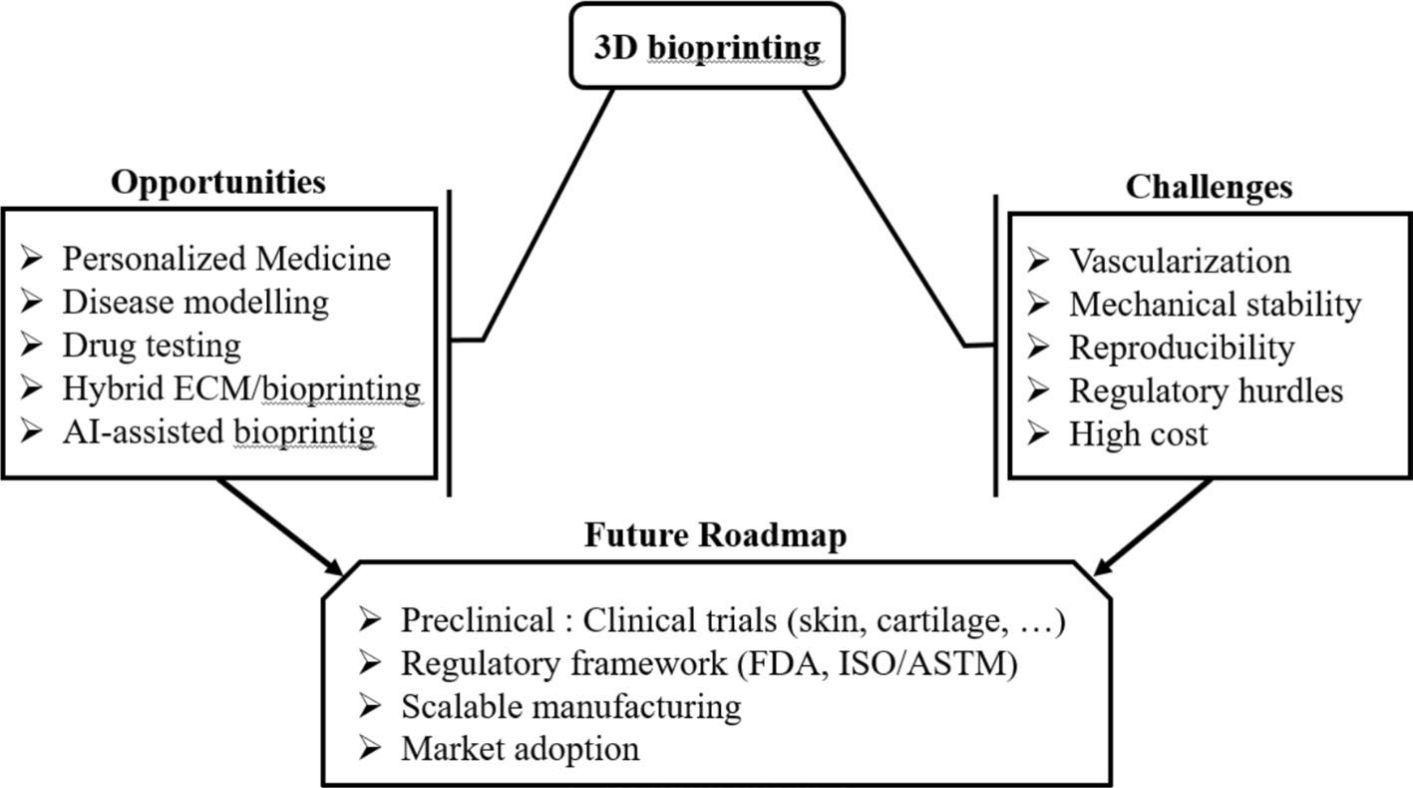
Conceptual diagram of opportunities, challenges, and the roadmap of 3D bioprinting

Despite these promising developments, significant challenges remain. The high cost of 3D bioprinting technology currently limits its use to well-funded research labs and hospitals, although the development of low-cost 3D bioprinting systems is underway. Critical unmet challenges include achieving scalable vascularization in larger tissue constructs, integrating AI-assisted design and control for improved precision, and developing standardized protocols to ensure reproducibility and long-term tissue functionality. In addition, microfluidic integration and automated biofabrication strategies are emerging as key approaches to address these limitations. Addressing these technological and practical barriers will be essential for translating 3D bioprinting from experimental research to widespread clinical applications, ultimately enabling the creation of functional, complex tissues and organs in a reliable and reproducible manner [139]. To provide a clearer overview, a conceptual overview of the main opportunities, key challenges, and the future roadmap of 3D bioprinting is presented in Fig. 7.

## Conclusion

3D bioprinting has advanced rapidly, evolving from proof-of-concept demonstrations to applications in drug testing, disease modeling, and early stage regenerative therapies. This review highlights that the most promising directions lie in personalized medicine and disease-specific constructs, which already provide clinically relevant insights and may soon complement or replace certain animal models. Compared to other 3D biofabricated models, such as spheroids or organ-on-chip systems, 3D bioprinted scaffolds offer precise spatial control over cell placement, tunable microarchitecture, and the ability to incorporate multiple cell types and biomaterials within a single construct. These features enable more physiologically relevant tissue models, enhanced reproducibility, and scalability, making bioprinted scaffolds particularly advantageous for disease modeling, drug testing, and regenerative applications. Hydrogels, particularly advanced and ECM-derived formulations, remain central to bioink design, while new functionalization strategies continue to expand their applicability.

Despite remarkable progress, several urgent challenges remain. Vascularization, immune compatibility, reproducibility across laboratories, and the scalability of fabrication processes still hinder translation into routine clinical practice. Moreover, regulatory acceptance of 3D bioprinted constructs as standardized preclinical tools has yet to be achieved. Addressing these issues will require coordinated efforts in materials innovation, bioprinter engineering, and the establishment of robust validation frameworks.

Looking ahead, focused directions for future research include the development of hybrid bioinks that combine synthetic strength with biological fidelity, integration of 3D bioprinting with organ-on-chip and microfluidic systems for dynamic disease modeling, and the use of artificial intelligence to optimize design and printing parameters. Clinically, skin grafts, cartilage replacements, and vascularized tissue patches appear to be nearest to translation, whereas whole-organ 3D bioprinting remains a long-term goal. By synthesizing technological advances with clinical imperatives, 3D bioprinting is poised to gradually but significantly reshape biomedical research and therapeutic strategies.

## Data Availability

No data sets were generated or analyzed during the current study.
